# On the Speech Properties and Feature Extraction Methods in Speech Emotion Recognition

**DOI:** 10.3390/s21051888

**Published:** 2021-03-08

**Authors:** Juraj Kacur, Boris Puterka, Jarmila Pavlovicova, Milos Oravec

**Affiliations:** 1 Institute of Multimedia Information and Communication Technologies, Faculty of Electrical Engineering and Information Technology, Slovak University of Technology in Bratislava, 2412 Bratislava, Slovakia; 2 Institute of Robotics and Cybernetics, Faculty of Electrical Engineering and Information Technology, Slovak University of Technology in Bratislava, 2412 Bratislava, Slovakia; boris.puterka@stuba.sk (B.P.); jarmila.pavlovicova@stuba.sk (J.P.); 3 Institute of Computer Science and Mathematics, Faculty of Electrical Engineering and Information Technology, Slovak University of Technology in Bratislava, 2412 Bratislava, Slovakia; milos.oravec@stuba.sk

**Keywords:** windows, frequency scales, spectrograms, psychoacoustic filter banks, LPC, cepstral features, phases, speech emotions, classification

## Abstract

Many speech emotion recognition systems have been designed using different features and classification methods. Still, there is a lack of knowledge and reasoning regarding the underlying speech characteristics and processing, i.e., how basic characteristics, methods, and settings affect the accuracy, to what extent, etc. This study is to extend physical perspective on speech emotion recognition by analyzing basic speech characteristics and modeling methods, e.g., time characteristics (segmentation, window types, and classification regions—lengths and overlaps), frequency ranges, frequency scales, processing of whole speech (spectrograms), vocal tract (filter banks, linear prediction coefficient (LPC) modeling), and excitation (inverse LPC filtering) signals, magnitude and phase manipulations, cepstral features, etc. In the evaluation phase the state-of-the-art classification method and rigorous statistical tests were applied, namely N-fold cross validation, paired *t*-test, rank, and Pearson correlations. The results revealed several settings in a 75% accuracy range (seven emotions). The most successful methods were based on vocal tract features using psychoacoustic filter banks covering the 0–8 kHz frequency range. Well scoring are also spectrograms carrying vocal tract and excitation information. It was found that even basic processing like pre-emphasis, segmentation, magnitude modifications, etc., can dramatically affect the results. Most findings are robust by exhibiting strong correlations across tested databases.

## 1. Introduction

Speech provides a natural and very complex form of communication as it can be rather precise by the means of grammar and it can convey side information about our state (physical, mental) and our attitude to what is said or to whom it is told (emotions). Such information is added to the speech rather unintentionally and humans can spot the smallest swings in their mood. When it comes to interaction with machines, these basic capabilities are not so easy to realize. As human machine interaction is becoming more human like, the speech emotion recognition (SER) is getting more important and has been an active research area for some time. SER systems have several applications and their usage is still widening. Such systems are used in call centers [1] prioritizing callers and assigning them to proper agents or in an online learning making the human–machine interaction more effective [2]. Moreover SER systems find their place in medical applications as well, e.g., [3], where the system helps military medics detect specific changes in soldiers’ behavior in early stages. A similar system was applied in 2010 in Haiti after earthquakes [4]. SER systems are also useful in automotive industry, where they can increase safety for drivers, e.g., in [5] an in-car conversation was analyzed, and in [6] visual emotion recognition was added making the system more accurate.

Emotions can be recognized from the so-called body language, face-play, and speech. Most of their characteristics are changing with age, education, experience, etc. Moreover, there is variability among speakers, their body language, and facial expressions. Therefore there are strong differences across ethnic groups, cultures, and even genders [7]. The exact detection of emotions is difficult and even an expert cannot classify emotions without any errors. In speech and perhaps other domains, it should be differentiated between natural and artificial emotions that can differ [8]. There is no objective definition for a single emotion. The basic, so called archetypal emotions according to [8] are anger, disgust, fear, joy, sadness, surprise, and neutral. In other research [9,10] different classes were used, e.g., boredom, hot/cold anger, approval, attention, slow/fast neutral, etc. In [8,11] authors showed that there is a strong dependence among some groups of emotions. If the amount of energy is regarded emotions like joy, anger, and fear fall into a high energy emotional class and sadness, boredom, and neutral to a low energy class. To distinguish between positive and negative emotions (joy and anger) with the similar arousal, the valence criterion was introduced.

For any speech emotion analysis it is important to understand the speech production process. Such process encodes different sorts of information and consists of four stages, i.e., thought (what to say, how, etc.), language (expressing thoughts by vocabulary and grammar, which introduces social background, education, etc.), prosody (stress, intonation, pace, etc., which is given by a learnt behavior, context, emotions, etc.), and air vibrations (caused by vocal organs reflecting physical personal features). As it can be seen emotions are spread over several stages. Having the physical signal it is necessary to know its basic characteristics that are: non-stationarity (stationary intervals for analyses range from 10 to 30 ms), frequency ranges (300 Hz to 4 kHz–speech recognition, and coding, 8 kHz–speaker recognition, and diagnostics), energy distribution (varies in time and frequency based on phonemes, e.g., vowels, fricatives, stops, etc., and can be up to 50 dB), and it is combined by an excitation signal (noise like or quasi periodic) and a vocal tract response (with dominant formant frequencies). However besides the speech production its perception is important too. Substantial for the detection of different sounds are [12]: number, location, and width of formant frequencies (vocal tract), intensity is perceived non-linearly, and frequency resolution declines with the frequency. On the contrary, irrelevant aspects are: overall tilt of spectra, i.e., X(ω)ω^α^, removal of frequencies below the first and over the 3 rd formant, and narrow band stop filtering. As speech contains a variety of information it is important for a particular application to extract specific features. The features are classified as acoustic (short time physical properties of vocal tract or excitation signal—related to an individual), prosodic (span longer time as stress, pace, pitch, etc.—identifying actual mood and learnt behavior), and higher level features (vocabulary and style—disclosing social background). Features can be further classified to parametric and non-parametric methods based on models they use and domain they operate in, e.g., time or frequency.

Therefore, as the recognition of speech emotions is a complex problem variety of approaches have been presented. The introduction of new machine learning approaches and the existence of large and complex databases caused a massive boom in this research domain. There are many articles elaborating on main aspects of SER systems, i.e., feature extraction and classification techniques. Most of them are based on a very successful deep learning approach. In [13] a comparison of different convolutional neural networks (CNNs) architectures can be found. The Mel based filter bank consisting of 40 filters covering 260 frames, i.e., 2.6 s was used as a feature vector (FV). It was shown that the application of different architectures provided small improvements to the success rate. A similar approach was presented in [14] using a 256 × 256 spectrogram as FV for CNN with 3 convolutional layers and 2 fully connected layers that was compared with a pretrained AlexNet network. It was found that using a pretrained CNN on a different dataset followed by a fine-tuning on a speech emotional dataset had no real effect on the recognition rate. Square kernels are the most preferable with CNN architectures; however, rectangular kernels were presented in [15], where each convolution layer had a different kernel size. The idea was based on the fact that rectangular windows could better cover the location of a single emotion in a spectrogram. The original spectrogram was split into the number of segments to achieve the proper simulation of continuous speech, similar to [13], and for better fine tuning of a complex CNN architecture. Different modifications of kernel types designed for SER systems were also tested, e.g., multi-convolutional kernels used across different convolution layers [16], or Fisher’s kernels [17].

Unlike many other research, the authors in [18] were focused on the speech signal processing, namely cyclostationary spectral analysis by which they estimated a second order spectral content using the Fourier transform accumulation method. There are many, more or less complex features that could be used in SER systems, e.g., in [19,20] a Teager energy operator (TEO) was used to dynamically adapt to various pitch and formants distributions to improve their sensitivity to emotional changes. The papers [21,22] were focused on time and speech segmentation aspects involved in SER.

Apart from CNN there are a few methods not being based on deep learning. In [23] the authors applied Gaussian mixture modeling (GMM) of a feature space. A FV was created by a combination of several prosodic features derived from fundamental frequency undergoing different modifications, Mel frequency cepstral coefficients (MFCCs), and linear predictive cepstral coefficients (LPCCs). Such FV, prior to the GMM modeling was subjected to a dimension reduction process. In [10] the authors researched on the Geneva minimalistic acoustic parameter set (GeMAPS) in affective computing. GeMAPS contains frequency related parameters (pitch, jitter, and formants), energy/amplitude related parameters and spectral parameters that formed a final FV consisting of 36 parameters. In addition to this, an extended parameter set was proposed using MFCC coefficients (from 1 to 4), spectral flux, and formant bandwidths (second and third). These parameter sets were evaluated both in arousal and valence dimensions. The authors in [24] used several low-level acoustic features, such as MFCC, eGeMAPS, and some high-level acoustic features produced by speech recognition networks as they have superior capability to extract speech related information. Then different classifiers, e.g., K-nearest neighbors, random forest (RF), and support vector machines (SVM) were applied to such FV. In [25] the authors extracted several features as MFCC, formant, and fundamental frequencies, on which different statistics were calculated. By doing so FVs having 166 coefficients were constructed. Consequently they were fed into a SVM based classifier. In [26] also a SVM classification method was applied, this time to a mixture of MFCCs, LPCCs, shifted delta cepstrum, and RASTA-PLP (perceptual linear prediction) features, where both one against all and one against one scenario were tested.

The feature extraction problem for SER is not yet fully solved. With the application of machine learning, and vast complex databases, different concepts have been introduced, e.g., autoencoder based ones or end-to-end processing. However, usually the more complex systems are, the less valuable information (knowledge) about the basic speech characteristics relevant for SER is available as it is hidden deep in the classification models. Therefore, in this paper the variety of basic speech characteristics, time, frequency, and energy aspects, signal processing methods, and their settings used for extracting basic features were analyzed by the means of machine learning. The goal was to find relevant methods and settings, disclose possible dependencies how particular settings especially in combination with others affect the accuracy, and augment the knowledge which basic speech characteristics and settings are important for SER systems and to what extent. Such extensive, detailed, and evaluated experiments should help to design a particular SER system in an effective way without an excessive tuning. There are many auxiliary features used in SER like: pitch, jitter, spectral slope, Hammarberg index, etc., that are derived from the basic ones, but still they can be mimicked by complex classification models applied to as versatile features as, e.g., spectrograms. Therefore spectrograms form the base for our further processing enabling direct access to most of the tested parameters. In addition to that CNNs were used as eligible and complex enough classification tools for their outstanding performance when processing image-like (spectrograms) features. The selected methods and settings were subjected to rigorous comparison based on statistical hypotheses testing using N fold validation and paired *t*-test. Finally, the robustness of some relevant findings was supported by correlating (Pearson and rank one) the results with another database, whereas performing hypotheses tests.

## 2. Materials and Methods

Regarding the main aim of the article, in the following a brief presentation of applied methods and considered speech characteristics is given.

### 2.1. Time–Frequency Distributions, Spectrograms

As stated before, it is important to analyze speech in time and frequency at the same time. There are several options how to do it, e.g., Wigner–Ville distribution [27], Wavelet transform [28], etc., but practically a short time discrete Fourier transform (ST DFT) is currently the most preferable approach for several reasons. It forms spectrograms when applied frame by frame to speech signals. Spectrograms carry both vocal tract and excitation signals, and magnitude and phase information as well. This makes them very flexible basic features suitable for further processing in many speech applications. Their importance is growing in connection with very complex modeling techniques and large datasets, where a more severe feature extraction may even deteriorate the overall performance. It is so because features are not optimal for given applications, i.e., may suppress vital information, whereas still preserving noise-like (missing SER related information) parts. The time-frequency resolution of spectrograms is controlled by the length of speech frames, their overlaps and applied windows. Usually a proper trade-off must be found considering a particular domain, application and the environment. More on frames, windows, DFT, and spectrograms in the speech processing area can be found, e.g., [12].

### 2.2. Auditory Filter Banks and Psychoacoustic Scales

Filter banks (FBs), especially auditory ones, are very popular tools for extracting relevant vocal tract information (spectral envelops). Their design follows different perception phenomena or auditory models. Psychoacoustics cover more aspects, therefore different scales and related FBs have been derived, depending on a certain phenomenon in focus, e.g., Mel FB (MFB) is based on a pitch perception, Bark FB (BFB) deals with the concept of critical bands, Gammatone FB (GFB) approximates an impulse response of human auditory system, etc. All the mentioned auditory FBs follow different aspects of a human perception, thus it is important to investigate, which are relevant and to what extent for the SER task; therefore they were incorporated in the experiments. More on FBs can be found in, e.g., [12,29], etc.

### 2.3. Linear Model of Speech Production

The most widely used model for speech production is a time variant linear model utilizing linear prediction coefficients (LPCs). The model combines an excitation signal with parameters of a vocal tract to form the speech signal. The vocal tract is represented by an all-pole filter that directly models formant frequencies, whereas modeling of zeros (nasal phonemes) is usually inefficient. Due to the computation process (mean square error) higher formants of lower energy can be neglected. Therefore it is common to perform a high-pass filtering (pre-emphasis) removing a low-pass filter effect of a sound propagating into an open space. Having model parameters and a speech signal, it is possible to estimate an excitation signal through an inverse filtering. Excitation signals mostly carry prosodic information, e.g., fundamental frequency, gain, and thus can be interesting also for SER systems. For more details on LPC refer to, e.g., [12,30].

### 2.4. Phase Information

The phase information is not used very often in speech analyses due to many reasons. However, in some articles [31] it was reported to improve the results. Construction of phase spectrograms poses two main difficulties: phase discontinuity along frequency and time axes. To suppress discontinuity along a frequency axis a phase unwrapping can be applied, which is ambiguous in a discrete case. To make it more robust a frequency up sampling by factor *M* can be used. Another source of a phase variability stems from processing time shifted frames. The aim is to compensate phases of periodic signals so that they would be constant regardless of the frame position, i.e., removing phase modifications caused by a processing of time shifted frames, while tracking their natural evolution comprised in real speech signals. A straightforward solution is the elimination of the well-known circular time shift property of DFT for each frame (*m*) as follows.

(1) $$\varphi \prime_{m}\left(k\right)=[\varphi_{m}\left(k\right)-\frac{2\pi kSm}{N}{]}_{2\pi },$$

where *S* is a frame shift, *k* is a frequency index, *N* is a frame length, *φ_m_* and *φ’_m_* are the original and compensated phases, and └ ┘2*π* is a modulo operation. However, we found this standard method cannot be directly applied in the case of up sampled frequency components (by factor *M*, i.e., there will be *NM* frequency components calculated from a frame of N samples). In such a case we derived frequency dependent compensation terms to be applied to the original signal *x*(*n*) prior to the DFT. The compensated signal *x_comp k_* is calculated as in (2).

(2) $$\begin{array}{c} x_{comp\;k\;}\left(n\right)\;=x\left(n\right)e^{\frac{-j2\pi kS}{NM}}\;\;\;\;\;\;\;\;\;n\;\epsilon \;\left\{S\ldots \;N-1\right\}\\ x_{comp\;\;k\;}\left(n\right)\;=x\left(n\right)e^{\frac{-j2\pi k\left(S-N\right)}{NM}}\;\;\;\;n\;\epsilon \;\left\{0\ldots \;S-1\right\} \end{array}$$

Then the up sampled DFT is applied to *x_comp k_*(*n*) of the length *N*, whereas producing *NM* frequency components. This secures the same phases across the time shifted frames covering all *MN* frequency components *k*. It should be noted that *x_comp k_*(*n*) is frequency depended and thus must be calculated for each frequency *k*.

Another option is to unwrap phases separately for each frame and for a fixed frequency, e.g., *k_ref_* set a constant phase (*φ_ref_*) to all frames. Finally, the phases of remaining frequency components (*k*) are adjusted as in (3).

(3) $$\varphi \prime_{m}\left(k\right)\;=\varphi_{m}\left(k\right)+k\left(\frac{\varphi_{ref}-\varphi_{m}\left(k\right)}{k_{ref}}\right)$$

### 2.5. Cepstral Features Using Psychoacoustic FBs (MFCC and GFCC)

Cepstral features are popular in the speech processing (speech and speaker recognition) and the most widely used is MFCC [12] that is based on MFB because of its good performance, compact representation, and easy computation. Its coefficients have clear representation, i.e., lower cepstral coefficients reflect low frequency changes, whereas the higher ones depict sharp changes in log spectra, and c0 represents log gain. They are efficient to suppress convolutional noises, separating components merged by convolution, etc. However, they are inefficient in handling additional noises. This concept can be generalized to different FBs, e.g., Bark or Gammatone, producing BFCC or GFCC features. More on cepstral features in speech processing can be found in [12].

### 2.6. Convolutional Neural Networks

Neural networks (NNs) [32] are applied in many areas, providing the best competition results in, e.g., face and speech recognition. It is due to the availability of huge training data, massive computational power, new structures, and enhanced training algorithms. For processing of 2D signals, e.g., pictures the most preferable structure is CNN [33]. It efficiently treats specific variabilities in the resolution and location while being able to extract many patterns in a hierarchical manner. The choice for CNN is obvious as speech related features (spectrograms, FBs) are assumed to exhibit such variations and patterns. Particular emotional patterns will be spread in time and may be located at any position, and due to speaker variability a certain shift may exist also on the frequency axis. The resolution variability comes from different pace of speaking (time axis) and location of formant frequencies (frequency axis) that is speaker dependent; no rotation like modifications are expected in the case of spectrograms or FBs.

**Figure 1:**
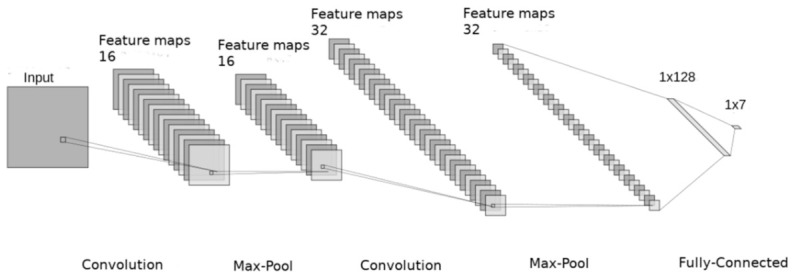
The structure of used convolutional neural network (CNN). Its input size changes to fit the size of feature vector (FVs) that varied across tests.

CNNs with 2 layers, consisting of 5 × 5 kernels (Figure 1) were deployed in the experiments, as they were successfully used for SER systems utilizing spectrograms. The first layer had 16 and the second one 32 kernels. Max-pooling layers of the size 2 × 2 were placed at the output of each convolutional layer. Consequently a fully connected layer with 128 hidden neurons followed by a dropout layer (retention probability 0.5) was deployed. Finally, there was a soft max activation layer classifying data into 7 classes, and ReLU (rectified linear unit) was chosen as an activation function. The CNN architecture was implemented in the TensorFlow backend [34]. A mini-batch size of 45 with a learning rate 0.01, Adam algorithm [35] for the training, and a categorical cross-entropy as a loss function were used. A validation loss was used as a metric for early stopping and data were divided into 15 folds. Each fold approximately consisted of the same number of recordings per emotion and was gender and speaker independent. The validation set accounted for 20% of random recordings of the training set for each fold.

### 2.7. Statistical Evaluation of Results

Numerous methods/settings deploying a single parameter statistical hypothesis tests were compared to see which are statistically better at a given significance level α. There are many measures whereas the most detailed one is the receiver operating characteristic (ROC) curve and its simplification—an area under ROC curve (AUC). These are too descriptive and rather computationally demanding (multiclass problem) and thus impractical in this large set of method-ranking experiments. Therefore it was decided to use very popular and rather representative measure of accuracy utilizing both true negatives and true positives samples defined as in (4).

(4) $$accuracy\left[\%\right]=\;\frac{\#True\;Positives+\#True\;Negatives\;}{\#all\;samples}\;100$$

To alleviate some of its drawbacks the classes having the same number of samples were secured across all the experiments. Even though different measures may differ in a value, naturally, they are highly correlated, e.g., for a 2-class same-sample-size problem and the least correlated scenario the correlation between the accuracy and F1-score was more than 0.91. Under some conditions measures may be even the same, e.g., accuracy and unweighted averaged recall (UAR) in a same-size scenario. Thus the selection of any proper measure should not substantially affect the ranking of methods, which is the main goal of the article.

*N* fold validation is used to compare two methods, the best one (*Mx*) with the highest average accuracy *µ_x_* against the remaining methods (*My* with *µ_y_*) in each experiment. The tested hypotheses were in the form:

(5) $$H_{0}\left(\mu_{x}\leq \mu_{y}\right)\;againts\;H_{1}\left(\mu_{x}>\mu_{y}\right)$$

It was checked if the best method *Mx* is actually equal or even worse to other methods with the maximal decision error equal to *α*. Methods *My* for which *H_0_* could not be rejected were regarded as statistically equal to the best one, whereas the decision error is at most α. As methods and settings were tested on the same data applied to the same folds, i.e., results were dependent, paired *t*-test [36] applied fold-wise was used. Methods based on accuracy intervals they fall in at some significance level were ranked in concluding remarks, i.e., following hypotheses were tested:

(6) $$H_{0}\left(\mu_{x}\geq Min\;accuracy\right)\;againts\;H_{1}\left(\mu_{x}<Min\;accuracy\right)$$

Finally, the least successful methods were tested similarly if they were equal to a random generator or were still able to extract some SER relevant information. As we had seven categories the tested hypotheses were:

(7) $$H_{0}\left(\mu_{x}=1/7\right)\;againts\;H_{1}\left(\mu_{x}\neq 1/7\right)$$

Fundamental to all tests based on a paired *t*-test is Gaussian distribution of data. In the case of 15 folds the Shapiro–Wilk test for small size data was applied. In most cases the results passed the Gaussianity test at he t10% significance level. For more details on statistical hypothesis testing see, e.g., [36].

To see how general the results are a different database was tested. Correlations (Pearson) [37] between accuracies of the 2 databases separately for each experiment and together were calculated as well. In addition to that a rank correlation (Spearman) [37] detecting any monotonic trend in the scorings of tested methods/settings observed on the databases was calculated for each test and in general. To see if the results-findings equally apply to different databases, the following hypothesis was tested:

(8) $$H_{0}\left(Z_{d1d2}=0\right)\;againts\;H_{1}\left(Z_{d1d2}\neq 0\right)$$

*Z_d_*_1*d*2_ is the Fisher transformation of Pearson or Spearman correlation coefficient (*ρ*) between accuracies of the 2 databases denoted as *d*1 and *d*2 and calculated as:

(9) $$Z_{d1d2}=1/2ln\left(\frac{1+\rho_{d1d2}}{1-\rho_{d1d2}}\right)$$

In the case of uncorrelated normally distributed accuracies *Z_d_*_1*d*2_ has an asymptotical normal distribution so the *H*_0_ (methods/settings observed on the databases are uncorrelated) was rejected if

(10) $$\left|Z_{d1d2}\;\right|>\frac{u\left(1-\alpha /2\right)}{\sqrt{N-3}},$$

*u* is a quantile of normal distribution, *N* is the number of samples, and α is the required significant level. Finally a match ratio (*Mr*) between equally performing methods (*EQ*) observed separately on the original and tested databases was calculated as in (11)

(11) $$Mr\left(d1,d2\right)=\;\frac{EQ\left(d1\right)\cap EQ\left(d2\right)}{EQ\left(d1\right)\cup EQ\left(d2\right)}$$

### 2.8. Databases

The Berlin database of emotional speech [38] was used in all experiments, whereas the IEMOCAP (Interactive Emotional Dyadic Motion Capture) database [39] was used to verify our findings. The Berlin database is in German and consists of 535 utterances with a 16 kHz sampling frequency. Ten different texts are spoken by 10 actors (5 men and 5 women) in the studio environment. The texts are independent from emotional categories: anger, disgust, fear, boredom, happiness, neutral, and sad. Recordings were labeled by 15 people to 7 emotional categories based on their subjective opinion. IEMOCAP contains recordings by 5 male and 5 female actors in 5 sessions with more categories. In the experiments only the corresponding classes were extracted using all actors to match the Berlin database.

## 3. Results

Several different aspects of speech signals (time, frequency, vocal tract, excitation, etc.) that are relevant for SER using many methods and settings were tested in the designed set of experiments. Therefore the following results were grouped into experiments focusing on a particular aspect.

### 3.1. Time and Segmentation

**Figure 2:**
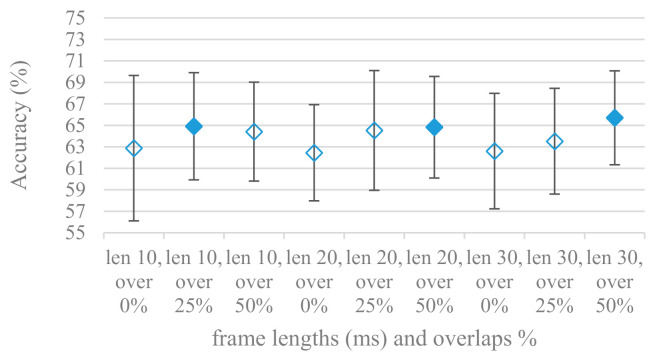
Averaged accuracies and standard deviations for 10, 20, and 30 ms frame lengths (len) with 0, 25, and 50% overlaps (over) and a 4 kHz frequency range. Filled marks show equally performing settings.

**Figure 3:**
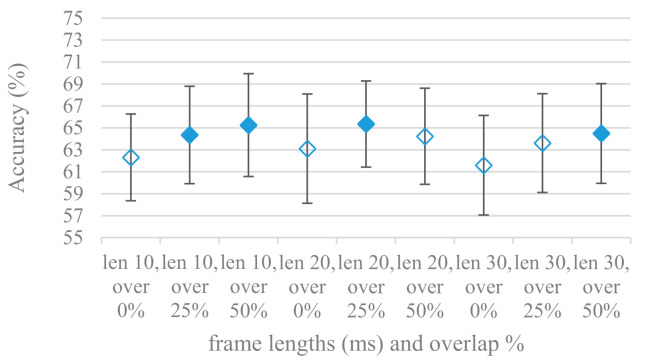
Averaged accuracies and standard deviations for 10, 20, and 30 ms frame lengths (len) with 0, 25, and 50% overlaps (over) and 8 kHz frequency range. Filled marks show equally performing settings.

Features that are common in SER applications using CNNs, i.e., power-based spectrograms spanning a fixed time of 1 s were used in time related experiments. At first the relation between accuracy and the applied segmentation, i.e., frame lengths and overlaps was tested. Frame lengths control a trade-off between time and frequency resolution while overlaps control the time resolution, amount of available training data and their redundancy. Average accuracies and standard deviations are shown in Figure 2 for frame lengths ranging from 10 to 30 ms combined with 0%, 25%, and 50% overlaps in the case of a 4 kHz maximal frequency. Filled marks show equally performing methods to the best one at the 10% significant level. The same is depicted in Figure 3 for an 8 kHz maximal frequency.

There is a visible trend in both graphs regarding the size of overlap, which is rather independent of the frame length, i.e., higher overlaps lead to better accuracies. This suggests the higher number of frames (higher overlap) in spectrograms that increase its time resolution and redundancy is beneficial for the CNN classification. On the other hand, the tested frame lengths were not dominant factors for the accuracy as all the lengths managed to record equal performance at the 10% significance level, i.e., the time-frequency resolution given by frame lengths in the tested range is satisfactory for SER.

**Figure 4:**
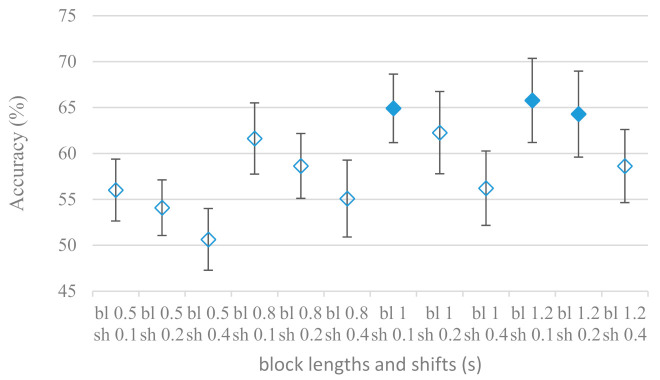
Averaged accuracies and standard deviations for 0.5, 0.8, 1, and 1.2 s block lengths (bl) with 0.1, 0.2, and 0.4 s shifts (sh) between adjacent blocks and an 8 kHz maximal frequency. Filled marks show equally performing settings.

The next experiment took into consideration lengths and shifts (complement to an overlap) of speech blocks that are used to form spectrograms, i.e., FVs for CNNs classification. The longer the block the more information is provided, however an extremely long speech part may potentially cover changes in emotions and reduce the amount of available training data. When considering the shift of adjacent blocks the larger the shift the less available blocks there are, but neighboring blocks are less redundant. The effect of spectrogram lengths (0.5, 0.8, 1, and 1.2 s) in combination with their shifts (0.1, 0.2, and 0.4 s) is shown in Figure 4 for an 8 kHz upper frequency; very similar results were recorded for a 4 kHz frequency limit. The 1.2 s upper bound for spectrogram lengths was given by the shortest recording in the database.

The result was obvious, the longer blocks (spectrograms in time) the better accuracies, however, the increase seemed to start to saturate above 1 s. The size of shifts between spectrograms acted reversely in all cases, i.e., the longer shift the worse accuracy. This means the amount of training data prevailed over the increased redundancy among FVs.

**Figure 5:**
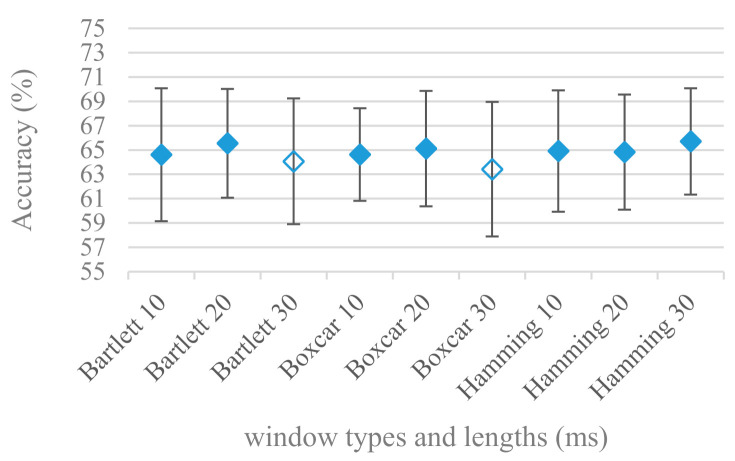
Averaged accuracies and standard deviations for Bartlett, Boxcar, and Hamming windows, in case of 10, 20, and 30 ms frame lengths, and 4 kHz maximal frequency. Filled marks show equally performing settings.

**Figure 6:**
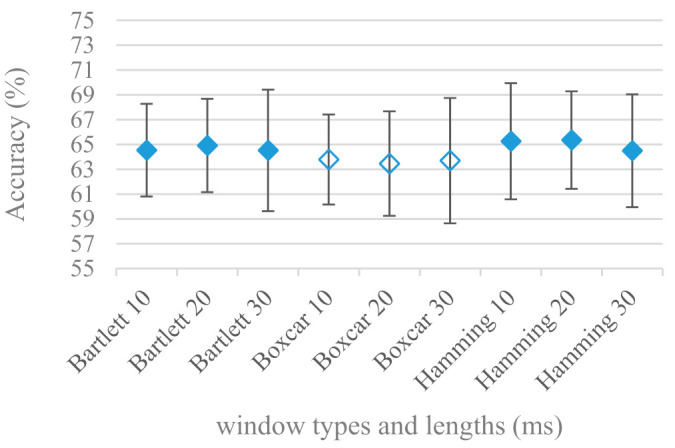
Averaged accuracies and standard deviations for Bartlett, Boxcar, and Hamming windows, in the case of 10, 20, and 30 ms frame lengths, and 8 kHz maximal frequency. Filled marks show equally performing settings.

The effect of applied windows, i.e., Boxcar, Bartlett, and Hamming in combination with different sizes of speech frames was considered in the last set of experiments involving segmentation. Figure 5 shows the results for the above-mentioned windows with lengths: 10, 20, and 30 ms, and a 4 kHz maximal frequency. The same experiment in the case of 8 kHz is shown in Figure 6. For the 4 kHz maximal frequency (Figure 5) the windows performed rather equally, however in the case of 8 kHz maximal frequency (Figure 6) the slow-decaying frequency characteristic of Boxcar caused a statistically significant drop in the accuracy regardless of the frame length. This may suggest that when considering higher frequency ranges the long tail aliasing is getting more disturbing for SER applications.

### 3.2. Frequency Ranges and Frequency Scales

**Figure 7:**
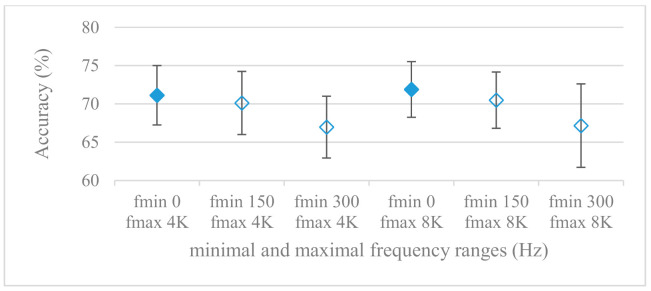
Averaged accuracies and standard deviations as functions of minimal and maximal frequency limits; Fmin {0+, 150, 300 Hz}, Fmax {4, 8 kHz}; and no pre-emphasis. Filled marks show equally performing settings.

**Figure 8:**
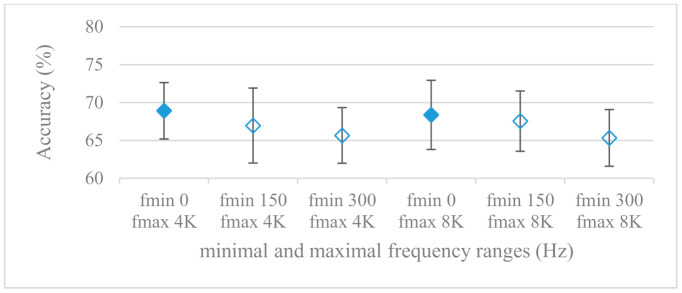
Averaged accuracies and standard deviations as functions of minimal and maximal frequency limits; Fmin {0+, 150, 300 Hz}, Fmax {4, 8 kHz}, and with pre-emphasis. Filled marks show equally performing settings.

This set of experiments used magnitude-based spectrograms. Accuracies for minimal (0^+^, 150, and 300 Hz) and maximal (4 and 8 kHz) frequency limits are shown in Figure 7. The effect of pre-emphasis is demonstrated in Figure 8. Notation 0^+^ Hz means the first frequency index after the unidirectional offset, thus the minimal frequency was determined by a particular frequency resolution. As it can be seen, the pre-emphasis actually deteriorated the results for all tested ranges. A remarkable observation was the positive effect of low frequencies under 300 Hz, which is in line with the failure of pre-emphasis. Moreover, the increase of the frequency range above 4 kHz seemed to be of minor additional benefit, especially in combination with pre-emphasis.

**Figure 9:**
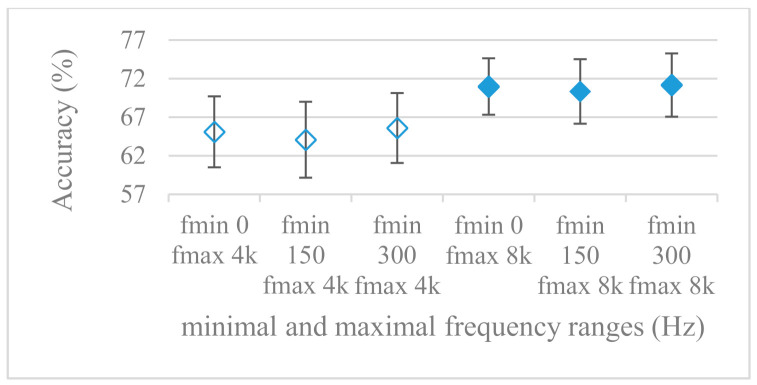
Averaged accuracies and standard deviations in case of Mel scale for minimal and maximal frequency limits; Fmin {0+, 150, 300 Hz}, Fmax {4, 8 kHz}, and no pre-emphasis. Filled marks show equally performing settings.

**Figure 10:**
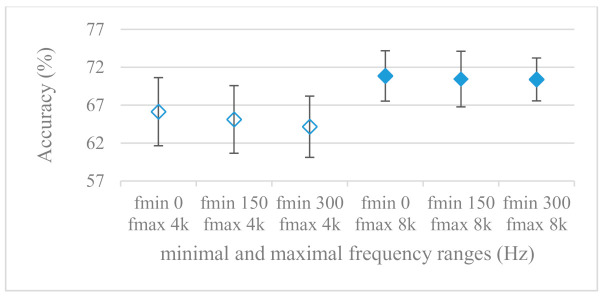
Averaged accuracies and standard deviations in case of Mel scale for minimal and maximal frequency limits; Fmin {0+, 150, 300 Hz}, Fmax {4, 8 kHz}, and with pre-emphasis. Filled marks show equally performing settings.

Consequently the deployment of a nonlinear frequency scale using the psychoacoustic Mel scale was investigated. The results without and with pre-emphasis and tested frequency ranges are shown in Figure 9 and Figure 10, respectively. In the same way as in the previous case the pre-emphasis slightly deteriorated accuracies for all settings. A notable observation is the significant improvement achieved by introducing the higher upper frequency limit, i.e., 8 kHz over 4 kHz in both cases. Nevertheless, this is caused by having more samples that (due to Mel non-linearity) provided a finer resolution for lower frequencies. However, if the linear frequency range is properly set, it slightly (on average) outperforms the Mel scale.

**Figure 11:**
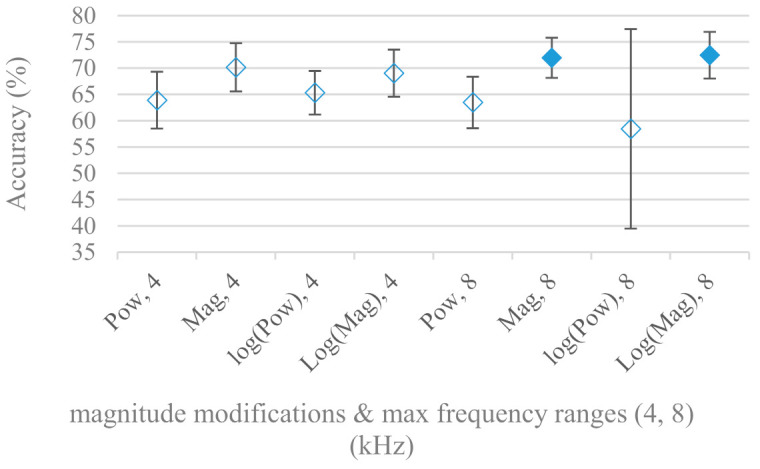
Averaged accuracies and standard deviations for different modifications of spectral magnitudes: magnitude (Mag), power (Pow), log magnitude, and log power, for 4 and 8 kHz maximal frequencies. Filled marks show equally performing settings.

The results varied a lot depending on the magnitude manipulation methods in the frequency range experiments. Therefore magnitude, power, log magnitude, and log power applied to spectrograms having different frequency ranges were tested. The results are shown in Figure 11 for 4 and 8 kHz ranges. As can be seen, the applied spectrum modification is an important factor depending on the frequency range that must be taken into consideration. Magnitudes scored significantly better regardless of the deployment of logarithm. On the other hand, powers and log powers were inferior especially when higher frequencies (8 kHz) were considered.

### 3.3. Vocal Tract Spectra Estimated by FB and LPC

**Figure 12:**
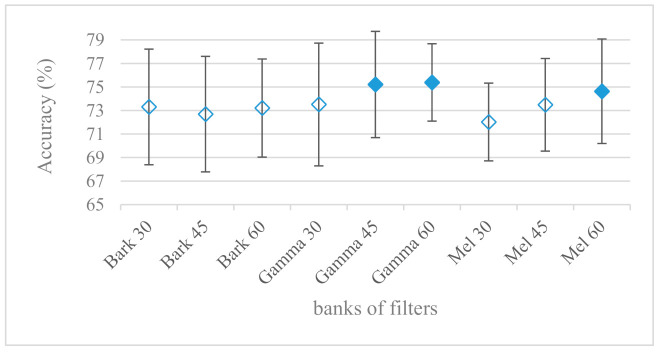
Averaged accuracies and standard deviations for Mel, Bark, and Gammatone FBs, having 30, 45, and 60 bands with an 8 kHz maximal frequency. Filled marks show equally performing settings.

So far, the signal was processed as a whole containing both excitation and vocal tract characteristics. Only spectral envelopes (vocal tract information) estimated by FB and LPC were examined in these experiments. The results for Mel, Bark, and Gammatone filter banks are shown in Figure 12 with different number of bands and an 8 kHz maximal frequency. The most successful were Gammatone FBs, however the Mel one cannot be rejected as inferior at the 10% confidence level. Furthermore, the higher number of bands had a positive effect, which is evident for Mel and Gammatone FBs.

**Figure 13:**
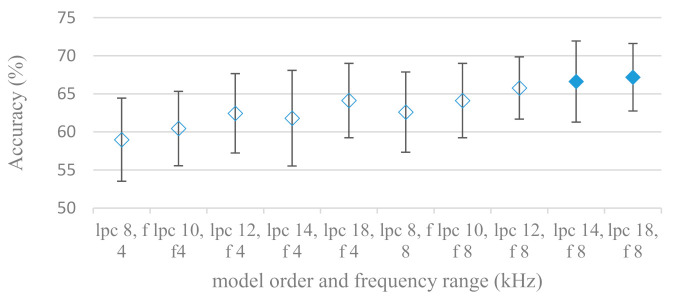
Averaged accuracies and standard deviations for linear prediction coefficient (LPC) model orders (lpc) ranging from 8 to 18, for 4 and 8 kHz maximal frequencies (f), and no pre-emphasis. Filled marks show equally performing settings.

**Figure 14:**
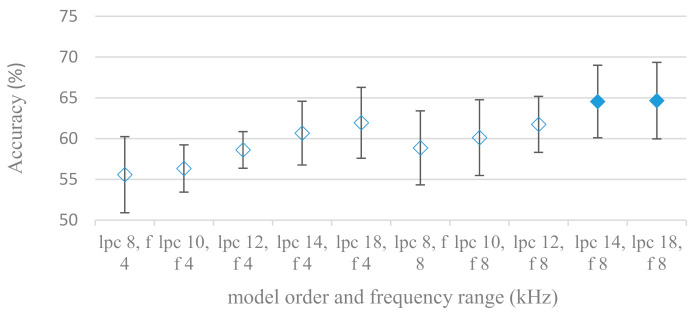
Averaged accuracies and standard deviations for LPC model orders (lpc) ranging from 8 to 18, for 4 and 8 kHz maximal frequencies (f), and pre-emphasis. Filled marks show equally performing settings.

**Figure 15:**
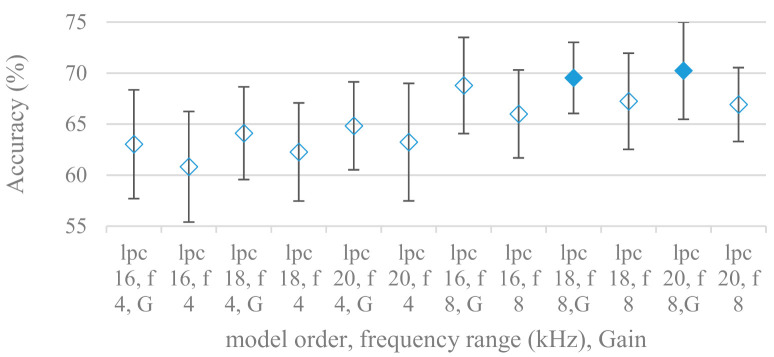
Averaged accuracies and standard deviations for LPC model orders (lpc) {16, 18, 20}, maximal frequency (f) {4, 8 kHz}, with and without gain (G), and no pre-emphasis. Filled marks show equally performing settings.

Consequently, spectrograms derived from LPC with different model settings were constructed. Effects of a LPC model order and a maximal frequency range are shown in Figure 13; without pre-emphasis. Results for the same settings using pre-emphasis are depicted in Figure 14. There is a clear trend, the accuracy increased with the model order and with the maximal frequency range. On the other hand, the pre-emphasis, which is a standard technique when performing LPC speech analysis, failed as in other cases. It should be noted that the listed results reflected only the shape of spectral envelops, i.e., the gain of LPC model was not included (as it is a part of excitation). Thus in the next experiment its deployment was tested and shown in Figure 15.

In all settings the incorporation of gain significantly increased the accuracy; the best results were recorded by model orders of 18 and 20 with gain and an 8 kHz maximal frequency.

### 3.4. Excitation Signal Spectra

**Figure 16:**
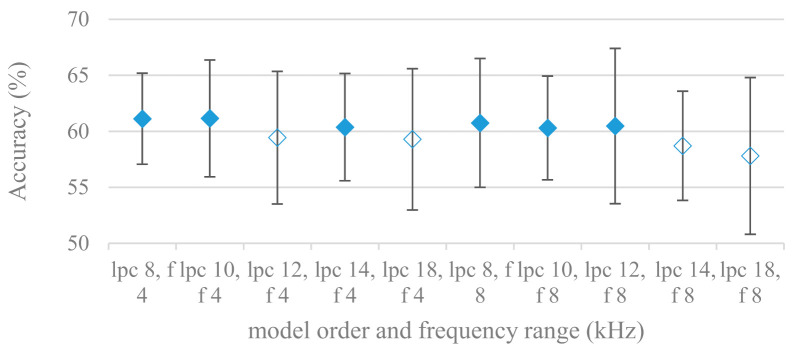
Averaged accuracies and standard deviations for spectrograms constructed over excitation signals with different LPC model orders (lpc) {16, 18, 20}, maximal frequencies (f) {4, 8 kHz}, and no pre-emphasis. Filled marks show equally performing settings.

**Figure 17:**
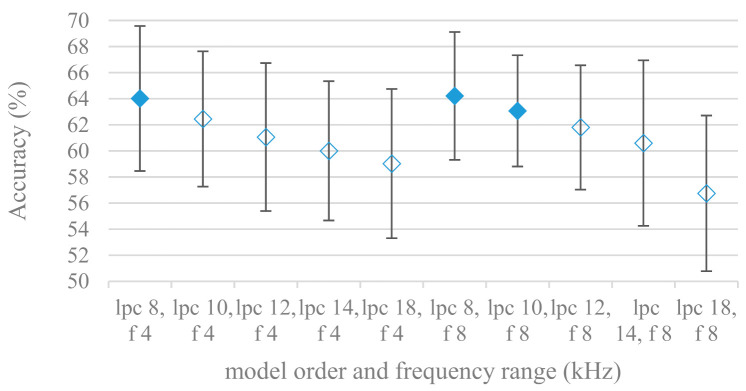
Averaged accuracies and standard deviations for spectrograms constructed over excitation signals with different LPC model orders (lpc){16, 18, 20}, maximal frequencies (f) {4, 8 kHz}, and pre-emphasis used to derive LPCs. Filled marks show equally performing settings.

The residual signal (excitation) to check also the effect of glottal and pre glottal features on SER was extracted from the LPC model. Model orders, maximal frequency limits, and pre-emphasis were tested as well. It should be noted that here the pre-emphasis (if used) was not applied to the excitation signal, but only to the computational process deriving LPC coefficients, which is a standard technique. The results for spectrograms constructed over excitation signals derived from different LPC model settings are shown in Figure 16. The same results are displayed with the application of pre-emphasis in the LPC calculation process in Figure 17. Compared to LPC related findings shown in Figure 13 and Figure 14, rather opposite observations can be seen in the case of excitation signals, i.e., pre-emphasis is clearly beneficial and in general the accuracy is not growing with the model order. It may suggest that the strategy of having less precise LPC model works. Such a filter (inverse) applied to speech does not filter out the spectral envelop thus more vocal tract components leak into the excitation signal. The pre-emphasis emphasizes higher frequencies and therefore the inverse LPC filter removes more high -frequency components from speech.

### 3.5. Phase Processing

**Figure 18:**
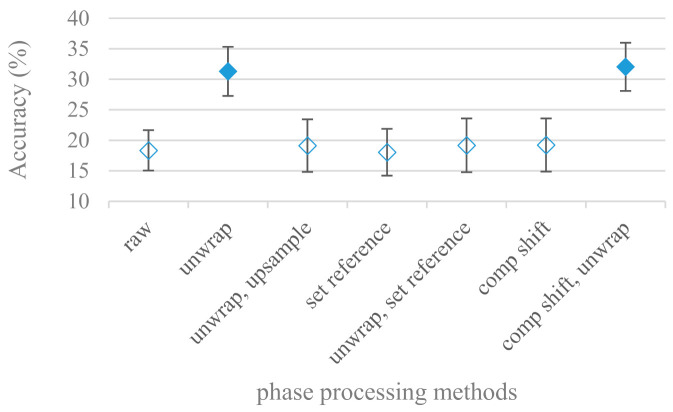
Averaged accuracies and standard deviations for phase spectrograms using following modifications: raw, unwrap, up sampled and unwrap, set reference phase, unwrap and set reference phase, shift compensation, and shift compensation and unwrap in the case of an 8 kHz maximal frequency. Filled marks show equally performing settings.

So far this paper was focused on magnitude spectra eliminating all phase information. Therefore in the following set of experiments the effect of phase signals on SER was considered. The results for the following phase extraction and modification methods were tested in Figure 18 in the case of an 8 kHz range: raw, unwrap, up sampled and unwrap, set reference phase, unwrap and set reference phase, shift compensation, and shift compensation and unwrap. The same results for a 4 kHz frequency range were very similar, thus not shown for the sake of brevity.

**Figure 19:**
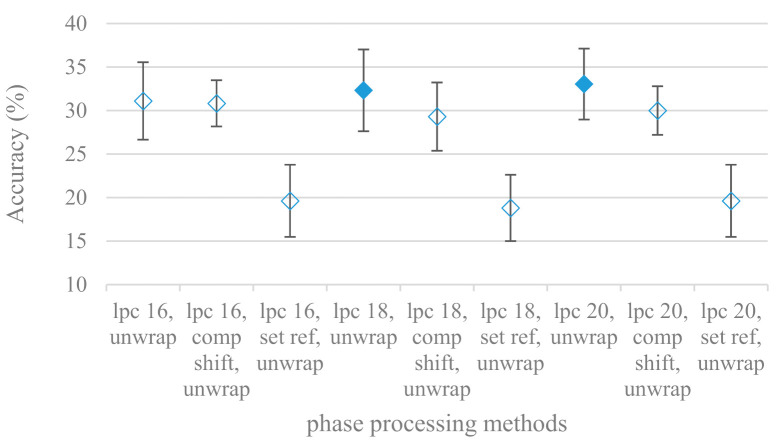
Averaged accuracies and standard deviations for phase spectrograms using following modifications: unwrap, unwrap and set reference phase, and unwrap and shift compensation in the case of an 8 kHz frequency limit and LPC model orders (lpc): 16, 18, and 20. Filled marks show equally performing settings.

Even though the accuracy was significantly lower, the phase unwrapping and unwrapping with the time shift compensation were significantly better than the remaining approaches. The phase experiments were extended also to excitation signals derived from an inverse LPC filtration. The most successful phase modifications, i.e., unwrap, unwrap and shift compensation, and unwrap and set reference were tested in combination with the following LPC model orders: 16, 18, and 20, and an 8 kHz maximal frequency are shown in Figure 19.

The best scores were recorded by unwrapping and unwrapping enhanced by shift compensation. The model order did not play any major role in the tested ranges. When compared to the speech signal as a whole the excitation based phases provided similar results.

### 3.6. Cepstrum Based Features

**Figure 20:**
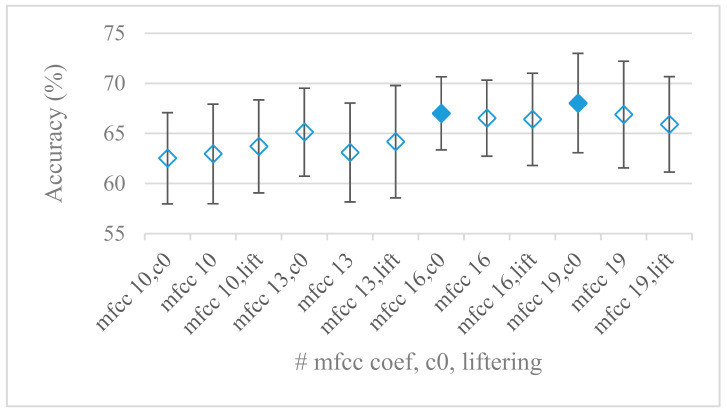
Averaged accuracies and standard deviations for the following MFCC settings: number of coefficients: 10, 13, 16, and 19, with and without c0, and liftering, for an 8 kHz maximal frequency and 60 FBs. Filled marks show equally performing settings.

**Figure 21:**
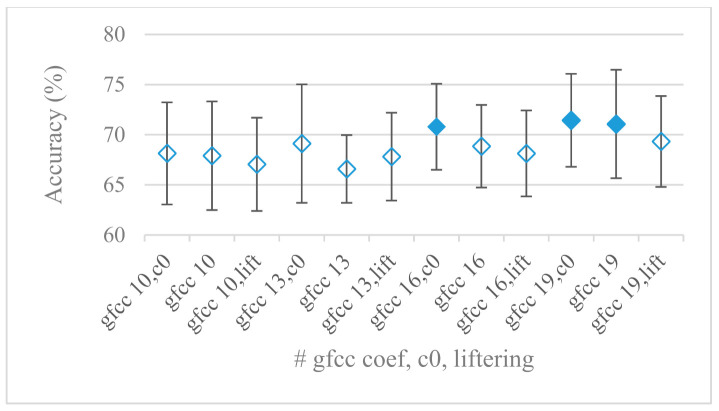
Averaged accuracies and standard deviations for the following GFCC settings: number of coefficients: 10, 13, 16, and 19, with and without c0, and liftering, for an 8 kHz maximal frequency and 60 FBs. Filled marks show equally performing settings.

Cepstrum based features that are more complex but very successful in many speech applications were analyzed in the last set of experiments. Different settings involved in the cepstrum calculation, i.e., liftering, *c_0_*, number of cepstral coefficients, in the case of an 8 kHz upper frequency and 60 filter banks (best settings for Mel and Gammatone FBs, Figure 12) were tested based on previous experiments. The performance of MFCC using 10, 13, 16, and 19 coefficients, with and without *c_0_* and liftering is shown in Figure 20. Referring to the promising results involving Gammatone FBs the construction of cepstral coefficients using Gammatone FB (GFCC) with the same settings as in the case of MFCC were tested as well. The results for GFCC are shown in Figure 21 and as in the case of Gammatone FBs, GFCCs scored better than MFCC. Though not very conspicuous but a trend of an increasing accuracy with the growing number of coefficients can be seen, e.g., the interval between 16 and 19 coefficients seems to work well. When considering the role of *c_0_* it proved to be beneficial in most cases especially with GFCC. On the other hand, the least successful was the liftering. MFCC consisting of only 35 FBs were tested just for curiosity. We realized such setting provides even better results, i.e., by 2% on average and by 2.8% in the best score compared to best performing MFBs. For the sake of brevity this experiment was not shown as the above-mentioned observations remain the same; naturally for 35 FBs the best number of cepstral coefficients decreased accordingly to 16.

### 3.7. Database and Classification Method Dependency Tests

So far all the listed results are based on the Berlin database. Tests on another database were performed to see how the methods/setting are consistent/robust. To keep the results succinct, correlations (Pearson) between accuracies on both databases for every experiment were computed. The ranking consistency of methods/settings between databases was evaluated by a rank correlation (Spearman) as well. Moreover a match ratio between sets of equally performing methods found in every experiment on both databases was calculated. Finally, the hypotheses that results/methods on different databases were uncorrelated to each other were evaluated at the 10% significant level. The results and average accuracies observed in each set of experiment are listed in Table 1. When the results were considered all together, the correlations between databases were as follows, Pearson correlation 0.25 (uncorrelation rejected), rank correlation 0.3 (uncorrelation rejected), and the match ratio of equally performing methods was 0.25. All correlations were positives, but in approximately 40% of the experiments the uncorrelated hypothesis can be rejected. However, even for low correlated results some common trends can still be inferred that will be discussed in the conclusions.

Finally, the choice of the classification method (CNN) for this task (spectrogram like FVs) was justified by comparing its performance on Berlin database for the best features (GBF experiments) with a SVM [40] approach (Gaussian kernel, slack variable, and one-over-rest scenario). On average CNN outperformed SVN by 45% (accuracy: 74.7% vs. 51.5%) and in the terms of the best scoring settings by 44.7% (accuracy: 75.38% vs. 52.1%). Moreover, the hypothesis on equality of both methods must be rejected at a 10% significance level.

### 3.8. Most Successful Methods and Settings

The methods and settings were grouped and ordered into proper accuracy intervals at the 10% significance level to summarize the results. Methods and relevant settings scoring over 75% and 70% accuracy ranges in the case of Berlin database are listed in Table 2. For the sake of brevity only 10 most successful methods falling into a 70% accuracy range are shown; in fact there were 28 methods in this range. The same results listing 5 best scoring methods observed on IEMOCAP database are shown in Table 3.

To see the most challenging emotions to differentiate between, a confusion matrix is shown in Table 4 for the best case and Berlin database. As it can be seen, the worst emotions to classify were happiness, which was mostly confused with anger and neutral misclassified with boredom.

## 4. Discussion

The presented series of results revealed some interesting findings that could be well generalized and these were discussed in more details as follows:

- As listed in Table 2, the most successful methods were derived from FBs using Gammatone, Mel, and Bark shape filters and scales. This means vocal tract features (spectral envelope) are very important, and the manner they are extracted and expressed, e.g., LPC model based features were proven to be statistically worse while extracting and representing the same sort of information. Even though the rank correlation is only 0.38, these findings mostly apply to the IEMOCAP database as can be seen in Table 3, i.e., the best methods are also GFB based.
- Other successful methods cover spectral based features (spectrograms) derived from the whole speech signals, i.e., containing both excitation and vocal tract signals. However, to do so they use significantly more parameters than FB features, e.g., 160 vs. 60, see Table 2. The settings proved to be important again, especially when frequency ranges in combination with magnitude modifying methods are regarded.
- The least successful methods were based on phase signals. However, neither of the tested settings failed, i.e., all were able to extract some SER relevant information so that the performance was significantly better than the one provided by a random classifier, i.e., 14.3% (seven classes). Thus they can still act as auxiliary features in some systems.
- In general the same ranking of methods, i.e., vocal tract based expressed by GFB, vocal tract and excitation signals using spectrograms and the least successful utilizing phases were observed on both databases. They however differ in some particular settings and ranges. This is mostly caused by differences in databases, e.g., by listening to some recordings it was noticed that the Berlin database consists mostly of genuine speech whereas in IEMOCAPS there were many recordings containing more background or speech artifacts. Thus some segments might not even contain a speech and a proper speech detection algorithm should be used. Moreover there was reported a significant difference between actors and native evaluators [37] in the perception of emotions for some scenarios.
- The time span of speech blocks (spectrograms) that were used for SER (classification) could significantly improve the accuracy. It is natural as more speech provides more valuable information. However, the increase becomes mild at around 1.2 s (Figure 4). The shift of adjacent speech blocks showed to be an important parameter as well, i.e., the shorter the shift the higher the accuracy. Nevertheless, this phenomenon is rather related to the increase of available data for CNN at the cost of increased redundancy across speech blocks. The same observations were made also for IEMOCAP database (rank correlation 0.7) however this time the shift (more data) was a more dominant factor.
- The effect of speech segmentation is documented in Figure 2 and Figure 3. As in the previous case longer overlaps (shorter shifts) of adjacent segments are preferred. However, in this case the bigger overlaps do not increase the number of training–testing samples (this effect is negligible) as the spectrograms are of a fixed time duration. It increases the number of frames in spectrograms, i.e., time resolution at the expense of increased redundancy, resulting in bigger FVs. The frame lengths (from which short time spectra were constructed) in the tested range were not a decisive factor, i.e., all lengths proved to be statistically equal (Figure 2). This means the frequency resolution resulting from 10 to 30 ms windows was adequate for SER.
- Windows types, e.g., Boxcar, Barttlet, and Hamming showed slightly different behavior depending on the used frequency range. For the 4 kHz upper frequency they acted equally from a statistical point of view (Figure 5). However, for the 8 kHz upper bound (Figure 6) Boxcar proved to be inferior in all cases. Boxcar is the least decaying window in the frequency meaning it causes frequency aliasing among distant frequencies. This probably proved to be more important for higher frequencies that got contaminated by lower frequencies having much higher energies. This phenomenon was not that critical in the 4 kHz limit as the “noise” from higher frequencies was presumably negligible compared to low frequency energies. Nevertheless this segmentation related setting in the tested ranges did not prove to be very decisive and robust as there was only negligible positive correlation (0.11) between databases.
- Very interesting and apparent was the effect of magnitude manipulation, i.e., power, magnitude, and logarithm of power and magnitude in connection with a frequency upper bound and the application of pre-emphasis. The magnitude processing is significantly dependent on the frequency ranges and pre-emphasis. Even though magnitudes scored better both for 4 and 8 kHz ranges (Figure 11), a quite substantial deterioration in the case of log energy and energy in combination with pre-emphasis was recorded for the 8 kHz range. These observations led to the following possible explanation. Higher frequencies are known to have rather small magnitudes for speech signals especially voiced ones. By the additional application of power these values get even smaller that is critical for a logarithm, e.g., if *X~R*(0,1) and *y* = *ln*(*x*), then *D*{*y*} = 12*D*{*x*} (*D* is dispersion), if power of *x* is used, the dispersion is further amplified by factor 4, i.e., *D*{2*ln*(*x*)} = 4*12*D*{*x*}. The application of a logarithm is a known practical problem, so to avoid singularities an energy threshold is applied, e.g., using a minimal positive number at a given precision. Nevertheless, high variations persist for low energies (high frequencies). Such amplified variations that are not caused by genuine speech naturally deteriorate the accuracy. This effect is crucial for spectrograms and vanishes when, e.g., a bank of filters are applied that average (dispersion reduction) energies over frequency bands. It should be noted that some of the deterioration may have been caused by the applied classification method; in our case CNN, which uses convolution (weighted combination of samples) and logarithm turns it into a multiplication of original samples powered to filter weights, which in general may be rather difficult to interpret. Thus the designers must be aware of this phenomenon and take into consideration magnitude modifications, frequency ranges, and pre-emphasis in connection with spectrograms and classification methods. An automatic application of logarithmic power, so common in speech processing, may not be optimal in such cases. Even though these phenomena show only a small positive correlation (0.07) between databases, a closer inspection of IEMOCAP results revealed that for an 8 kHz frequency range, all except a magnitude spectra modification, i.e., log power and log magnitude recorded a dramatic accuracy drop from approximately 40% to only 19%. So, this is strictly in line with the observations and explanations inferred from the Berlin database.
- While testing frequency ranges a positive effect of low frequencies, less than 300 Hz was found (Figure 7 and Figure 8). Likewise, higher frequencies, i.e., 8 kHz recorded better scores (Figure 11). It differs from the speech recognition systems that mostly operate in the 300 Hz to 4 kHz range. However, these systems aim to extract minimal lexical information, which increases their robustness in real environments. The benefit of a higher frequency limit (over 4 kHz) is in the line with our previous research on speaker recognition [41]. It shows the speaker specific information is also located in higher frequencies. Nevertheless, these findings favoring the (0+, 8 kHz) range hold true for clean recordings, and taking into account the previous discussion, the effect of an upper bound significantly depends on magnitude modifications when spectrograms are regarded. The positive effect for 0+ lower bound was confirmed also for IEMOCAP database (correlation 0.43), however the higher frequency bond had a slightly adverse effect compared to the Berlin database.
- When testing linear and nonlinear frequency scales in the construction of spectrograms (Figure 9 and Figure 10), it was observed that on average the linear scale slightly outperformed the non-linear Mel scale both for 4 and 8 kHz frequency limits. A non-linear scale may disrupt the clear trace of a fundamental frequency in a spectrum that is presented by sharp minima located at integer multiplications of a fundamental frequency. The course of fundamental frequency in time is known to reflect speech prosody, which is definitely controlled by emotions. On the other hand, the Mel scale significantly improved the performance for the 8 kHz frequency limit, which was not observed for the linear scale. Such a behavior can be attributed to the increased precision for lower frequencies over the higher ones (nonlinear scale) as the amount of frequency samples (FV size) in both cases was kept the same. It should be noted that the experiments regarding the Mel scale were highly correlated (0.92) to the IEMOCAP database.
- The importance of vocal tract characteristics extracted by the LPC model is presented in Figure 13, Figure 14 and Figure 15. It was clearly shown that the best performance was achieved by high model orders (18, 20) without a pre-emphasis. This focuses the model to low frequencies and a finer description of speech spectra. Nevertheless, the main contributing factor was the incorporation of gain that represents the speech dynamic, which is related to prosody and thus to emotions. In comparison to psychoacoustic FBs this method proved to be inferior. This can be due to model limitations, e.g., sort of an excitation signal, the model order, parameter estimation (the mean square error of a prediction signal), numerical problems, etc. A high correlation (0.93) between the databases for LPC and gain modeling was recorded.
- Excitation signals were also incorporated in our study as they carry relevant information about the speech prosody. They were derived from an inverse LPC filter of different orders. Interesting though explainable behavior was observed, i.e., the less precise the LPC model was the better results were. The best scoring one was with pre-emphasis (did not work for spectral envelopes) having only eight prediction coefficients. Based on the empirics related to LPC, a model order of 8 is not adequate for a precise modeling of speech sampled at 16 kHz. Naturally by having a less precise model more vocal tract information is leaking through an inverse LPC filtering into the excitation signal. This leads us to the conclusion that the improved results (for a less precise model) might have been a consequence of additional information resulting from an improperly removed spectral envelope. These findings are moderately correlated (0.4) to the IEMOCAP database.
- In a series of phase related experiments (Figure 18 and Figure 19) its modifications, e.g., unwrapping, upsampling, shift compensations, setting reference frequency to a fixed phase, etc., were tested. They were applied to both speech and excitation signals. In all cases the results were much worse (approximately by 50%) than the magnitude based approaches. Nevertheless, the most successful methods for phase extraction were unwrapping and unwrapping combined with time shift compensation.
- The most common feature used in many speech applications, i.e., cepstral coefficients in forms of MFCC and GFCC were tested in the concluding experiments. Based on the presented results (Figure 20 and Figure 21) the higher number of coefficients (16–19) led to better accuracies. The Gammatone based design outperformed the Mel one measured both on average and in the best scores. In almost all cases the incorporation of c0 (energy) was clearly beneficial, which is in line with expectations (prosody). However, when it comes to liftering, no obvious merit was observed. It is so because CNNs can easily and more precisely perform such operation if needed using its convolutional layers; moreover, it helps in adverse conditions to increase robustness for the sake of precision, which was not the case. MFCC and GFCC are more complex features involving many underlying settings bound in a nonlinear way. Thus finding optimal settings and making more general conclusions would involve a parallel optimization of all relevant parameters at the same time, e.g., a significantly worse Mel FBs using 35 bands outperformed the best one having 60 FBs (Figure 12) when it was subsequently used for the MFCC calculation. Still such results could not be easily extended to other MFCC/GFCC systems if some settings must be changed not even mentioning different background conditions. MFCC/GFCC experiments proved a solid consistency as they reached high correlations (0.87) between the databases.
- The selected CNN based classification was compared with another common approach—the SVM in the case of the best scoring methods and settings. The original choice proved to be superior for spectrogram- like processing over SVM where more than a 40% improvement was observed for the best features (GFB).
- The most challenging emotions to separate were happiness, which was mostly confused with anger and neutral misclassified with boredom. It is of no surprise as these emotions are very close to each other when considering acoustic and prosodic features. An obvious improvement to separate these cases would be to use high level speech features, e.g., vocabulary, phrases, etc., to get also a context of speech.
- While trying to remove a phase shift (in a frequency domain) between adjacent frames and the up sampled case (by factor M, to get more precise phase unwrapping), we found that the classical formula does not work. By a closer inspection we derived frequency dependent compensation terms that must be applied to the original signal (Equation (2)) prior to the DFT. By doing so the adjacent frames, after the DFT, will have their phases unaffected by the time shift, i.e., the distortion of phase characteristics caused by the time shift of *S* samples will be suppressed and only the natural (speech related) changes will be preserved.
- In the next work it should be analyzed, which features and settings affect a particular emotion, how successful speech features of different levels work together, and how they are related to other emotional modalities, e.g., facial features.

Nevertheless, the abovementioned findings still carry some issues such as:

- The databases consist of only acted emotions by professional actors that might not reflect their true emotions. So, the SER system and its settings only reflect emotions that we believe are correct based on our perception.
- The databases contain only clean speech recorded in the controlled environment. Thus the results do not include session variability, noise, etc., so the presented methods and settings may differ for mismatched conditions. Even though some of the main findings are rather robust as the cross database correlations on different levels were high.
- Even though CNN is a very complex classification method with outstanding results in image/speech processing, we cannot reject that the findings released here can be partly affected by its limitations (structure, training algorithm, numerical precisions, etc.). Nevertheless, when compared to another successful classification method—SVM, still the CNN provided better results.
- The experiments and outcomes are related to single language SER systems (German and English). However, in [42] it was shown that the performance of SER systems and features applied in a cross lingual scenario may drop substantially.
- Even though the knowledge of how each setting, method and preprocessing affects a SER system is important from the theoretical point of view, and may dramatically shorten the design of a particular SER system, in [43] it was documented that using complex classification models and databases it is possible to process even raw speech signals, while achieving challenging results. However, in such a case the extracted features (local or global) are hidden deep in the complex model structure.

Relation of the study to the existing research:

- There are many articles presenting particular SER systems focusing on new features, feature fusions, or modeling/classification methods in order to achieve the best performance. In contrary to that, there are few articles testing and analyzing the behavior of specific features and their settings in given conditions, e.g., testing frequency ranges or scales [44], etc. Nevertheless, published results measured even on the same database vary a lot, e.g., from approximately 50% [45] to even 92% [46], mainly due to the experimental set up, evaluation, processing, and classification. This study differs as it provides a unified, complex, and statistically rigorous analysis of great variety of basic speech properties, features and their settings, and calculation methods related to SER, by means of the machine learning. Where it was possible a generalization and explanation of particular findings was provided using signal theory. Some of the results were tested on another database to reveal their robustness. The presented results ranging from approximately 60% to 75% were in line with other similar systems. No obvious contradiction to other comparable results was observed. Nevertheless, differences attributed to the stochastic nature could be spotted, e.g., in [44] the Mel scale performed better (by 1.9%) than a linear frequency, whereas our results showed slightly better scores (by only 0.2%) in favor of a linear frequency, etc. Therefore we introduced and used the concept of equally performing methods throughout the result section.
- When viewing the results in terms of confusion matrices, i.e., ranking of emotions according to their accuracies, very similar outcomes were observed, e.g., in [47,48]. An interesting fact is that there was a perfect match with at least five other articles, where happiness recorded the worst scores and disgust, neutral, and boredom were also low-scorers. The situation with the best performing emotions was not that clear, however, fear and anger were amongst the best. This is also in line with our outcomes.

## 5. Conclusions

A wide set of experiments targeting basic speech signal characteristics, and speech processing methods applied to SER were deigned and evaluated. These experiments were designed to disclose how and to what extent the characteristics and methods are important for SER and help us to understand their meaning in this task. In some cases a high degree of generalization was observed using two databases. The most successful methods were based on vocal tract signals represented by filter banks (Gammatone, Mel, and Bark) covering the 0–8 kHz frequency range. Promising base features are also spectrograms carrying both vocal tract and excitation information processed by CNN. It was further shown that even such common processing steps like segmentations, windowing, pre-emphasis, and application of different magnitude modifying methods, e.g., power, log power, intensity, etc., can dramatically affect the results especially in combination with frequency ranges. Thus all of these common steps must be of high concern even if they are only parts of more complex features and scenarios. All the detailed and evaluated experiments should serve as practical and theoretical bases for constructing more robust, accurate and more complex SER systems.

## Figures and Tables

**Table 1:** Correlations of methods/setting across databases for each set of experiments.

Tests	Accuracy Correlation	*H*_0_-Accuracies Uncorrelated	Methods’ Rank Correlation	*H*_0_-Rank Uncorrelated	Match of Equally Performing Methods	Averaged Accuracy Berlin Database	Averaged Accuracy IEMOCAP Database
LPC modelling	0.93	F	1	F	1	0.7	0.35
Mell scales ranges	0.92	F	0.89	F	0.6	0.63	0.38
MFCC and GFCC	0.82	F	0.87	F	0.67	0.67	0.39
Classification block/spectrogram lengths	0.65	F	0.70	F	0.33	0.59	0.37
Linear frequency ranges	0.43	T	0.30	T	0.25	0.62	0.39
Filter banks	0.35	T	0.38	T	0.33	0.67	0.41
Excitation signals	0.4	T	0.3	T	0.6	0.6	0.39
Frame lengths/types	0.11	T	0.03	T	0.06	0.64	0.39
Magnitude modifications	0.07	T	0.09	T	0	0.66	0.33

T—hypothesis *H*_0_ is true (accepted), F—hypothesis *H*_0_ is false (rejected); Mel frequency cepstral coefficients (MFCC); gammatone frequency cepstral coefficients (GFCC).

**Table 2:** Methods and settings being in 75% and 70% accuracy ranges at the 10% significance level sorted in descending order (Berlin database).

Features	Fmin (kHz)	Fmax (kHz)	Magnitude Modification	Frequency Scale	Feature Vector Length	Signal Type: Excitation/Envelop	Accuracy (%)
Methods and settings in over 75% accuracy range							
GFB	0	8	mag	RBF	60	enve	75.38
GFB	0	8	mag	RBF	45	enve	75.21
MFB	0	8	mag	MEL	60	enve	74.63
GFB	0	8	mag	RBF	30	enve	73.51
10 best methods and settings in over 70% accuracy range							
MFB	0	8	mag	MEL	45	enve	73.48
BFB	0	8	mag	BAR	30	enve	73.3
BFB	0	8	mag	BAR	60	enve	73.2
BFB	0	8	mag	BAR	45	enve	72.68
Spect	0	8	Log mag	Hz	160	both	72.45
MFB	0	8	mag	MEL	30	enve	72
Spect	0	8	mag	Hz	160	both	71.88
GFCC, c0	0	8	Log pow	RBF	19	enve	71.4
Spect	0.3	8	mag	Mel	155	both	71.17
Spect	0	4	mag	Hz	80	both	71.12

Gammatone FB (GFB); Mel FB (MFB).

**Table 3:** The 5 best methods and settings observed on IEMOCAP database sorted in descending order.

Features	Fmin (kHz)	Fmax (kHz)	Magnitude Modification	Frequency Scale	Feature Vector Length	Signal Type: Excitation/Envelop	Accuracy (%)
GFB	0	8	mag	RBF	60	enve	44.35
GFB	0	4	mag	RBF	45	enve	44.24
GFCC	0	8	Log pow	RBF	19	enve	43.67
GFCC, c0	0	8	Log pow	RBF	19	enve	43.24
MFB	0	8	mag	BAR	60	enve	43.02

**Table 4:** Confusion matrix for 7 emotions, best settings, and berlin database (%).

Emotions	Fear	Disgust	Happy	Angry	Sad	Bored	Neutral
Fear	90.3	4.3	2.2	2.2	1.1	0	0
Disgust	10.6	75.2	1.2	0	0	10.6	2.5
Happy	16.7	1.9	44.4	36.1	0	0	0.9
Angry	1.4	0	3.5	95.1	0	0	0
Sad	2	6.9	0	0	79.3	10.3	1.5
Bored	0.6	2.9	0	0.6	4	85.5	6.4
Neutral	3.2	3.2	4.8	0	0	24.8	64

## Data Availability

The datasets (Berlin database of emotional speech and IEMOCAP) supporting the conclusions of this article are available at http://emodb.bilderbar.info/docu/ (accessed on 6 March 2021) and https://sail.usc.edu/iemocap/iemocap_release.htm (accessed on 6 March 2021). In a part of our experiments we used TensorFlow platform available at https://www.tensorflow.org/install? (accessed on 6 March 2021) under Apache License 2.0, implemented in Python 3.5 programming language.
